# A lightweight network model designed for alligator gar detection

**DOI:** 10.1038/s41598-024-61016-3

**Published:** 2024-05-08

**Authors:** Xin Wang, Wei Shi, Rong Chen

**Affiliations:** 1 Ningxia University, School of Information engineering, Yinchuan, 750021 China; 2 Lanzhou Vocational Technical College, School of Information engineering, Lanzhou, 730070 China

**Keywords:** Mathematics and computing, Information technology

## Abstract

When using advanced detection algorithms to monitor alligator gar in real-time in wild waters, the efficiency of existing detection algorithms is subject to certain limitations due to turbid water quality, poor underwater lighting conditions, and obstruction by other objects. In order to solve this problem, we developed a lightweight real-time detection network model called ARD-Net, from the perspective of reducing the amount of calculation and obtaining more feature map patterns. We introduced a cross-domain grid matching strategy to accelerate network convergence, and combined the involution operator and dual-channel attention mechanism to build a more lightweight feature extractor and multi-scale detection reasoning network module to enhance the network’s response to different semantics. Compared with the yoloV5 baseline model, our method performs equivalently in terms of detection accuracy, but the model is smaller, the detection speed is increased by 1.48 times, When compared with the latest State-of-the-Art (SOTA) method, YOLOv8, our method demonstrates clear advantages in both detection efficiency and model size,and has good real-time performance. Additionally, we created a dataset of alligator gar images for training.

## Introduction

**Figure 1:**
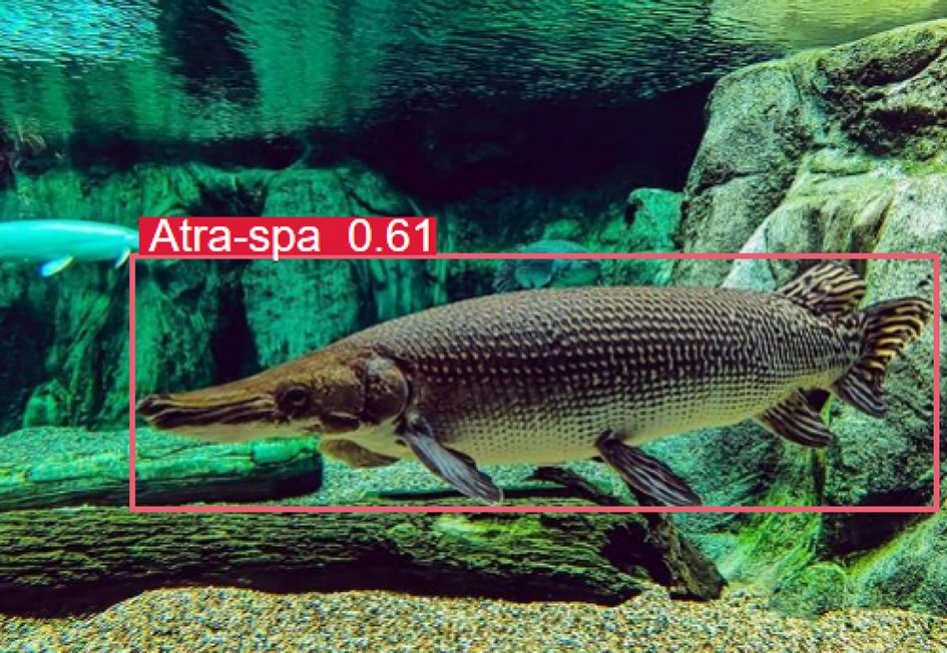
Alligator gar target detection example.

The alligator gar^1^, a carnivorous invasive fish native to North America, possesses a robust constitution, characterized by tough skin and sharp teeth, rendering it remarkably adaptable. Notably, this species exhibits an omnivorous dietary inclination, indiscriminately consuming a wide array of prey within its aquatic habitat. Regrettably, the introduction of alligator gar into natural aquatic ecosystems has led to widespread ecological disruption, often culminating in detrimental consequences for entire ecosystems and even posing a threat to human safety. Furthermore, it is worth emphasizing that the digestive tracts and eggs of the alligator gar contain highly toxic compounds, rendering them unsuitable for human consumption and devoid of significant practical utility. In recent times, there has been a surge in ecological security incidents attributable to the actions of the alligator gar, largely precipitated by human intervention. To mitigate such occurrences, we advocate for the deployment of submerged surveillance equipment within areas of suspicion, coupled with the implementation of real-time detection algorithms. This integrated approach facilitates the continuous monitoring of alligator gar activities, ultimately enabling effective real-time positioning and capture, as elucidated in Fig. 1.

Prior to the widespread adoption of deep learning technology, several target detection methodologies rooted in artificial models were employed for the identification and localization of alligator gar. These methods encompassed diverse approaches, each possessing its own set of advantages and limitations. Sliding Window-based Methods^2,3^: These methods involved the use of a fixed-size window that traversed the image, followed by a classifier to ascertain the presence of the target. While straightforward, this approach entailed significant computational demands, rendering it inefficient. Image Segmentation-based Methods^4,5^: These techniques initiated the process by segmenting the image into multiple regions, subsequently performing target detection within each segmented region. While capable of precise target localization, this approach relied heavily on high-quality image segmentation. Feature Extraction-based Methods^6,7^: Feature extraction methods operated by extracting image attributes such as color, texture, and shape, employing classifiers for target detection. This approach was effective in distinguishing targets from the background but necessitated meticulous feature selection and extraction. Template Matching-based Methods^8,9^: Utilizing a set of target template images, these methods matched them with the input image to identify the most similar regions as targets. While simple and expedient, they were sensitive to changes in target shape and scale. Machine Learning-based Methods: These methods encompassed machine learning techniques, including Support Vector Machine (SVM)^10^, Decision Trees^11^, Random Forests^12^, among others. This category leveraged trained classifiers to learn target features for detection. While capable of automatic feature learning, they exhibited limitations in managing extensive datasets and complex scenes. Despite their utility, these methods exhibited certain constraints related to target positioning accuracy, computational efficiency, and adaptability to complex environments. However, in recent years, the burgeoning development of deep learning has ushered in a new era for target detection. Approaches grounded in convolutional neural networks, such as Faster R-CNN^13^, YOLO^14^, SSD^15^, and others, have emerged as prominent contenders in the domain of target detection. These methods have not only achieved remarkable results but have also excelled in terms of accuracy and efficiency, firmly establishing themselves as the prevailing paradigm within the realm of target detection.

Numerous one-stage target detection algorithms^16–18^ have exhibited the capability to fulfill the requisites of alligator gar target detection. Among these, the YOLO (You Only Look Once) series of deep learning algorithms stands as a prominent exemplar, renowned for its capacity to concurrently predict the locations and categories of multiple targets within a single forward pass. A defining characteristic of these algorithms is their capacity to achieve accelerated real-time detection by reframing object detection as a regression problem, obviating the need for multiple sliding windows or region proposal networks applied to the image. YOLOv1^19^, the inaugural iteration of the YOLO series, emerged in 2016. It partitioned the image into multiple grids, forecasting both the bounding box and category of the target within each grid cell. These bounding boxes encompassed the target’s location (comprising center coordinates, width, height) and category probabilities. YOLOv1 boasted swift detection speeds but may have faced challenges in detecting smaller targets with precision. YOLOv2^20^, also recognized as YOLO9000, arrived in 2017 as the second iteration. It introduced several enhancements over YOLOv1, such as the utilization of more anchor boxes to handle targets of diverse shapes, multi-scale training to accommodate varying target sizes, and the incorporation of convolutional layers for feature extraction. Additionally, YOLOv2 introduced predictions for bounding box confidence and category prediction confidence, ameliorating detection accuracy. YOLOv3^21^, released in 2018 as the third iteration, further refined the architecture based on YOLOv2. It introduced a feature pyramid layer and multi-scale prediction, deploying three grids of different scales for prediction to better address targets of varying sizes, achieving a harmonious balance between accuracy and speed. YOLOv4^22^, the fourth iteration, launched in 2020. It introduced an array of technical enhancements, including an expanded network architecture, more convolutional layers, and higher-resolution inputs, aimed at further enhancing both detection accuracy and speed. These innovations, however, resulted in increased computational complexity. The YOLO series of algorithms have garnered widespread adoption across various applications owing to their real-time performance and robust detection accuracy. These applications span intelligent surveillance, autonomous driving, industrial inspection, and beyond. Each succeeding version within the series builds upon its predecessor to cater to diverse requirements and evolving challenges. Diverging from the trajectory of YOLOv4, YOLOv5^23^ adopts a more lightweight network structure, optimizing detection speed by curtailing parameters and computational load. While YOLOv4 introduced the Spatial Attention Module (SAM)^24^ and Path Aggregation Network (PANet)^25^ to enhance feature representation capabilities, YOLOv5 prioritizes lightweight design and real-time performance, while maintaining a requisite level of accuracy. This renders it particularly attractive for applications necessitating swift object detection, such as real-time surveillance and mobile deployments. Nevertheless, the detection of alligator gar in natural waters presents unique challenges. The underwater environment in untamed aquatic habitats is characterized by complexity, often featuring scenarios where alligator gar and other objects or species may obstruct and overlap with each other. Furthermore, alligator gar may appear in densely concentrated formations, yet monitoring imagery may capture only partial body segments. Fluctuations in the distance and angle between the swimming alligator gar and the monitoring lens can result in diminished proportions of the target within the image frame. Moreover, underwater monitoring equipment may capture distorted images due to the refractive properties of turbid water and suboptimal underwater lighting conditions. These factors collectively amplify the complexities associated with character identification and alligator gar detection. To the best of our knowledge, research pertaining to alligator gar detection utilizing the aforementioned advanced methodologies remains relatively limited.

To realize real-time detection of the alligator gar species, we have devised a real-time detection network model named ARD-Net (Alligator gar Real-time Detect Net). ARD-Net possesses a range of distinctive features designed to optimize its performance, as delineated below:

- Cross-Neighborhood Grids Matching Strategy: ARD-Net incorporates a cross-neighborhood grids matching strategy, which serves to augment the acquisition of positive sample anchor boxes. By doing so, it expedites the convergence process, enhancing the efficiency of the network’s training.
- Multi-Scale Detection Inference Network Module: A pivotal component of ARD-Net is the introduction of a multi-scale detection inference network module. This module leverages a dual-channel attention mechanism^26,27^ and involution^28^ operation to concentrate its focus on target objects of varying dimensions. Remarkably, it achieves this with a more streamlined parameter configuration, thereby elevating the network’s overall detection performance.
- Innovative Involution Structure: In acknowledgment of the prevalent challenge posed by redundant calculations within high-dimensional feature layers^29^ , ARD-Net introduces an involutional structure. This innovation extends to the construction of a lightweight feature extraction backbone network, thereby mitigating the model’s inference time and reducing the overall parameter count.

**Figure 2:**
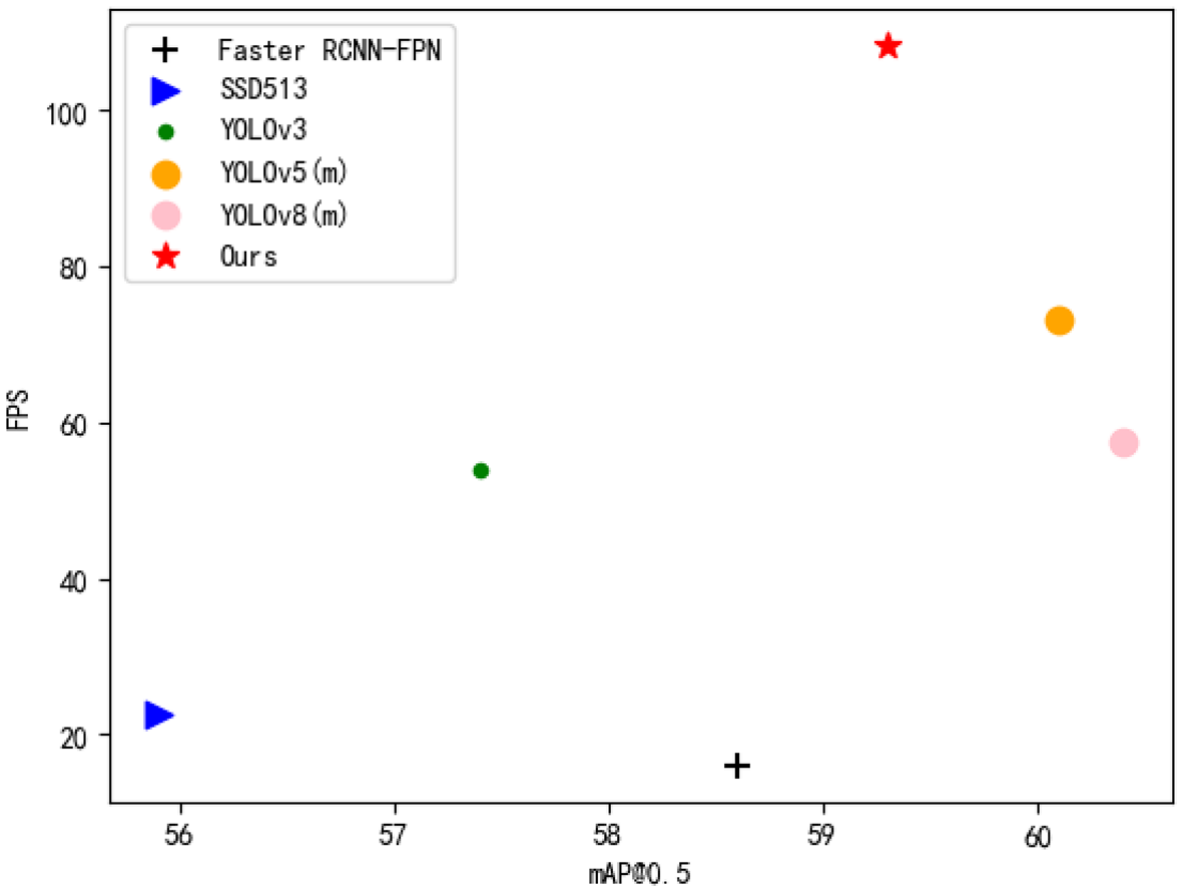
Illustrates a comparative chart, showcasing the mean Average Precision (mAP) and Frames Per Second (FPS) metrics for various detection methods.

Through these strategic enhancements, ARD-Net is engineered to enable real-time detection of the alligator gar species, effectively navigating the complexities presented by the aquatic environment and contributing to the conservation efforts in safeguarding indigenous aquatic ecosystems. Furthermore, we have meticulously curated a comprehensive dataset comprised of alligator gar images, which serves as the foundational resource for training our detection model. Empirical investigations conducted within our study have conclusively demonstrated that, in comparison to YOLOv5 and YOLOv8^30^, our method excels in multiple dimensions. Specifically, our approach not only boasts swifter real-time detection capabilities and a reduced model size but also showcases commendable performance in terms of detection accuracy and overall efficiency, as visually depicted in Fig. 2. ARD-Net is recognized as the inaugural model designed for swift target detection, specifically aimed at thwarting the encroachment of the alligator gar species.

In the ensuing section, we embark on a comprehensive exploration of our network model, intricately combining it with the detection mechanism inherent to YOLOv5. Subsequently, we provide an exhaustive exposition of the experimental findings, leaving no detail unexplored. Lastly, we engage in a discourse concerning specific scenarios where our approach may exhibit variances or limitations, offering a holistic perspective on its applicability and effectiveness.

## Related work

Object detection algorithms grounded in deep learning have demonstrated outstanding efficacy within the realm of computer vision, gaining widespread utilization for tasks related to detecting and recognizing objects in both images and videos.

### Faster R-CNN (region-based convolutional neural network)

The Faster R-CNN stands as a seminal two-stage target detection algorithm, incorporating a Region Proposal Network (RPN) to generate candidate regions. Subsequently, a convolutional neural network is employed for the dual purpose of classifying and performing bounding box regression on these identified candidate regions. The Faster R-CNN is capable of end-to-end generation of candidate regions and target detection, demonstrating commendable accuracy and stability. Nevertheless, it is encumbered by a relatively sluggish inference speed, primarily attributed to its two-stage design and the computational overhead associated with the Region Proposal Network (RPN).

### SSD (single shot multibox detector)

SSD is an algorithm employing multi-layer convolution for target detection. It anticipates bounding boxes and category information across various scales and levels, thereby enhancing the detection performance for objects at different scales. One drawback of SSD lies in its relatively diminished detection performance concerning small targets. This limitation arises from the prediction on multi-level feature maps, potentially causing information loss for smaller targets. Additionally, the anchor box design in SSD may necessitate manual adjustments to align with diverse datasets and target scales, introducing a level of complexity.

### RetinaNet

RetinaNet addresses the issue of category imbalance in target detection through the introduction of a specialized loss function named Focal Loss. This innovative approach enables effective handling of a substantial number of background categories, ensuring the maintenance of high detection accuracy. However, a notable drawback of RetinaNet lies in the potential difficulty in addressing class imbalance problems during training, particularly when dealing with extremely imbalanced object detection datasets. While the introduced Focal Loss proves beneficial in managing a large number of easily classified background samples, in certain extreme cases, additional adjustments and optimizations may be necessary.

The YOLO series stands as a pioneering one-stage real-time target detection algorithm, which reformulates the target detection challenge into a regression problem. This paradigm divides the image into fixed-size grid cells, where each cell undertakes the prediction of a bounding box, object category, and the confidence associated with the box. Presently, YOLOv5 and YOLOv8 represent the mainstream versions of this series. YOLOv5 places emphasis on detection efficiency and a lightweight model size, while YOLOv8 prioritizes higher detection accuracy.

### YOLOv3

YOLOv3 (You Only Look Once version 3) represents a real-time target detection algorithm, achieving simultaneous detection of multiple targets by partitioning the image into grid units. In each unit, the algorithm predicts multiple bounding boxes and their corresponding object categories. Utilizing feature maps of three different scales for detection, YOLOv3 exhibits a commendable blend of high detection accuracy and real-time performance, making it widely applied in target detection tasks for both images and videos. Nevertheless, YOLOv3 displays relatively diminished detection performance for small targets, and its capability to process overlapping targets in complex scenes may be constrained. Furthermore, the use of fixed-size grid cells impairs the spatial accuracy of YOLOv3 for targets compared to some Anchor-based methods.

### YOLOv5

YOLOv5, a target detection framework developed by Ultralytics, employs single-scale detection and leverages a lightweight backbone. It enhances small object detection by introducing the Feature Pyramid Network (FPN) to extract multi-scale features. The framework supports various data enhancement techniques, contributing to improved model generalization. YOLOv5 strategically seeks a balance between real-time performance and accuracy. In comparison to YOLOv4, YOLOv5 places greater emphasis on the lightweight and fast reasoning of the model while maintaining a high level of accuracy. However, YOLOv5 relies on extensive training sets and may encounter limitations in processing overlapping targets within complex scenes. Additionally, certain special tasks may necessitate further customization and adjustment. Despite its efficacy, YOLOv5 involves a significant number of parameters in its operations, leading to redundant calculations during training or reasoning on data samples. Consequently, for devices with limited resources, the model may exhibit slight bloat, leaving room for ongoing lightweight optimization.

## Methods

Leveraging insights derived from the YOLOv5 research, we have engineered the ARD-Net-a real-time detection model meticulously designed for alligator gar detection. ARD-Net represents a fusion of cutting-edge technologies, combining the power of deep convolutional networks^31^ with a multi-scale detection framework^32^. Additionally, it integrates the involution operator and the dual-channel attention mechanism, culminating in an efficient and high-performing architecture tailored specifically for alligator gar detection in real-time scenarios. Trained on our extensive alligator gar dataset, ARD-Net has demonstrated superiority over numerous alternative methods, particularly in terms of detection efficiency. In the subsequent sections, we will delve into a detailed exposition of ARD-Net, providing readers with a comprehensive understanding of its architecture, components, and the methodologies that underpin its exceptional performance.

**Figure 3:**
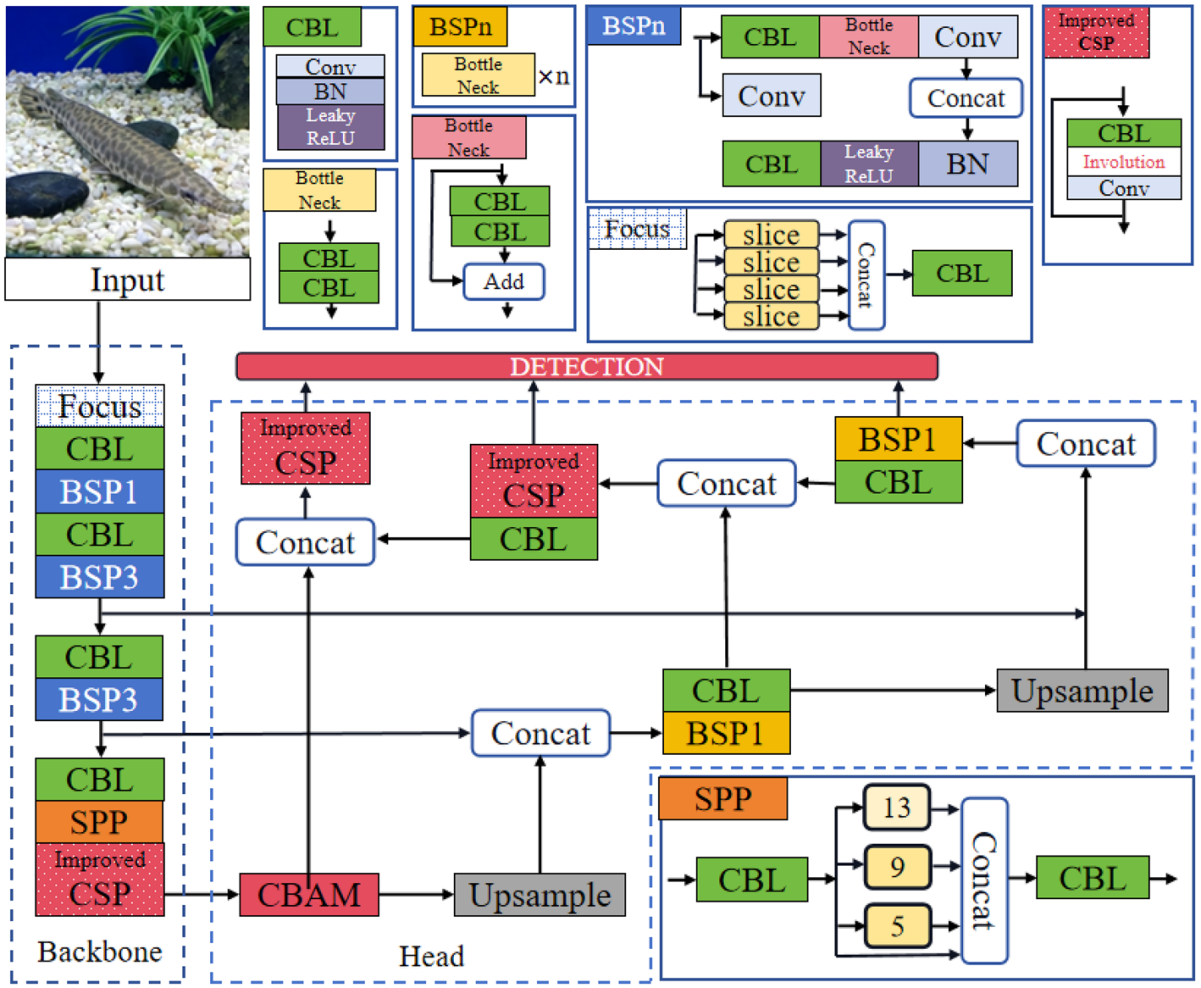
Schematic diagram of the ARD-Net network model structure.

The comprehensive structure of the network model is visually represented in Fig. 3, encompassing two primary components: the Backbone and the Head. The Backbone’s principal role is to extract image feature information, while the Head is responsible for generating detection results across varying scales. For detailed information on each component, please refer to the annotated diagram and the rest of this section. In a general comparison with the YOLOv5 structure, the most significant distinction of this model lies in the incorporation of the inner convolution module. This module proves advantageous in scenarios where the feature information of the network model yields higher-dimensional channel output, facilitating a substantial reduction in redundant calculations between channels. This, in turn, diminishes computational load, minimizes the model’s output size, and produces feature maps with diverse visual patterns. Consequently, the model becomes adept at effectively detecting alligator gar under varying lighting conditions, diverse water qualities, and other obstructive factors.

### Target box regression and cross-neighborhood grid matching strategy

**Figure 4:**
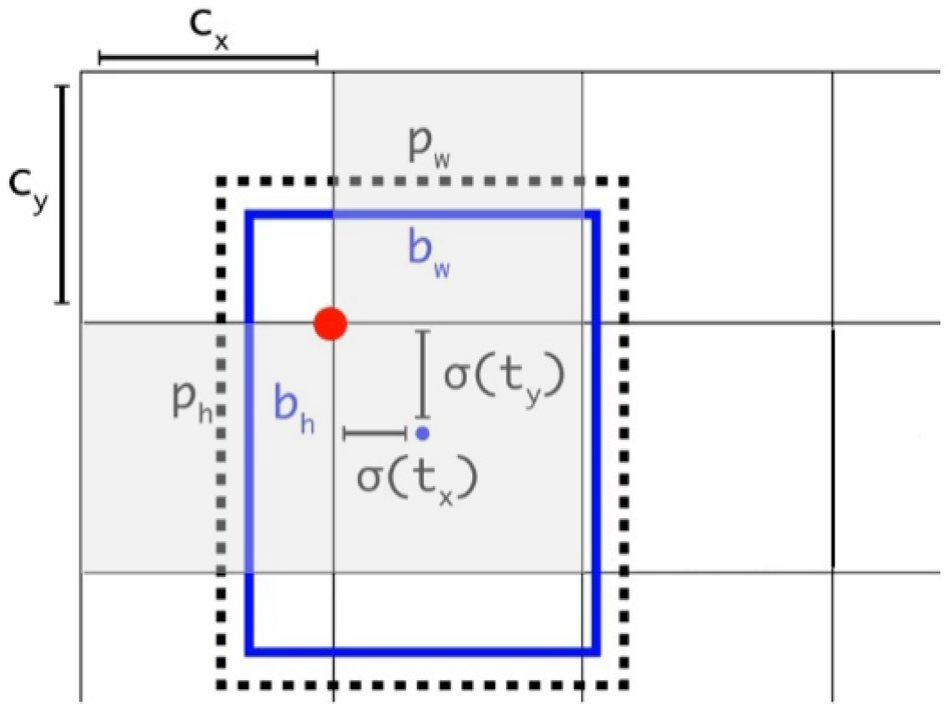
Target box regression and cross-domain grid matching.

In our target prediction output, we have adopted a marking method that corresponds to the methodology employed in YOLOv5. This involves utilizing an anchor box set to predict the bounding box parameters for each target. Each prediction box comprises essential information, including: Category Value, Center Point Coordinates, Width and Height of the Target. To accurately predict the bounding box, we perform regression to determine the offset between the actual width and height of the target and the predefined anchor box. The offset values are then normalized using the sigmoid function^33^. This normalization process^34^ ensures that the offset values fall within a suitable range, facilitating precise localization and sizing of the detected targets within the image. This normalization step, which involves applying the sigmoid function, restricts the value of the offset to fall within the range of 0 to 1. This restriction is crucial to prevent issues such as the loss function gradient going out of control^35^ and the loss value becoming NaN during the network’s learning and training process. By keeping the values within this bounded range, we ensure the stability and convergence of the training process, enabling the network to learn effectively. Assuming that the four offsets of the predicted bounding box correspond to the coordinate values $t_x$, $t_y$, width $t_w$, and height $t_h$, Fig. 4 elucidates the relative relationship between the predicted bounding box and the predefined anchor box. This visual representation offers a clear understanding of how the predicted bounding box is adjusted in relation to the anchor box through the offset values ( $t_x$, $t_y$, $t_w$, $t_h$).

1 $$\begin{aligned} \begin{aligned} \left\{ \begin{array}{l} b_x=2\sigma (t_x)-0.5+c_x, \\ b_y=2\sigma (t_y)-0.5+c_y, \\ b_w=p_w(2\sigma (t_w))^2, \\ b_h=p_h(2\sigma (t_h))^2. \\ \end{array}\right. \end{aligned} \end{aligned}$$

Indeed, the parameters involved in this matching operation serve distinct roles in localizing the detected target. Here’s a breakdown of their significance:

- $b_x$ and $b_y$: These values represent the actual coordinate values of the detected target’s center point.
- $c_x$ and $c_y$ denote the horizontal and vertical differences between the upper-left endpoint of the grid where the predicted target is currently situated and the origin (0,0) of the image. These values anchor the predicted bounding box within its respective grid.
- 0.5: This constant value, 0.5, represents the center point of the anchor box.
- $b_w$ and $b_h$ correspond to the actual width and height of the target, respectively.
- $p_w$ and $p_h$ denote the width and height of the predicted bounding box, respectively.

To ensure that the center point of the bounding box aligns with the current grid and to constrain all these parameters to an appropriate range, $t_w$, $t_y$, $t_w$ and $t_w$ undergo a normalization process through the $\sigma$ function. This normalization ensures that the predicted offset values fall within the range of 0 to 1, promoting stability and convergence during the network’s training process and facilitating accurate localization of the detected target. For instance, when $t_x=t_y=t_w=t_h=0$, then $\sigma (t_x)=\sigma (t_y)=\sigma (t_w)=\sigma (t_h)=0.5$, and $b_x=0.5+c_x$, $b_y=0.5+c_y$, $b_w=p_w$, $b_h=p_h$. Under this circumstance, the predicted bounding box aligns perfectly with the predefined anchor box, indicating a precise detection. As per equation (1), it’s evident that since both *q* and *w* have maximum values of 1, this mechanism effectively constrains the actual size of the predicted target bounding box to within a range of 4 times the dimensions of the preset anchor box. This level of control ensures that the predicted bounding box remains within a reasonable and manageable size relative to the anchor box, contributing to the stability and accuracy of the detection process. Furthermore, cross-neighborhood grid matching strategy is instrumental in acquiring a greater number of positive sample anchor frames, thereby expediting the convergence process during training.

### Efficient lightweight feature extraction paradigm: involution

The involution operator can be seen as a general case of the self-attention mechanism. However, it is more concise than the self-attention mechanism. Unlike the relatively fixed filtering mode of convolution, involution takes pixel values or areas in the feature map as input and generates a corresponding involution operator. It not only has a greater ability to perceive contextual correlations between pixels but also automatically adjusts the kernel size to match the input feature map size in the domain. According to a certain pixel of the feature map, The involution operator generated by the kernel generation function $\Phi$ for a specific pixel of the feature map can be expressed as $\mu _{i,j}\in R^{H\times W\times K\times K\times G}$. Here, *H* and *W* represent the height and width of the feature map, *K* is the size of the generated operator, and *G* represents the number of shared involution operator groups. If *C* denotes the number of channels in the feature map, the output feature information of the involution operator can be expressed as:

2 $$\begin{aligned} Y_{i,j,k}=\sum _{(u,v)\in \Delta k }\mu _{i,j,u+\left\lfloor K/2\right\rfloor ,v+\left\lfloor K/2\right\rfloor ,\left\lceil \llceil kG/C \right\rceil \rrceil }X_{i+u,j+v,k}. \end{aligned}$$

The involution operator can also be represented in a more generalized form:

3 $$\begin{aligned} \hfil \mu _{i,j}=\Phi (X_{\Psi _{i,j}}) \end{aligned}$$

where $\Psi _{i,j}$ represents the set of neighboring coordinates of pixel (i, j), and $X_{\Psi _{i,j}}$ is the tensor space encompassing the neighborhood surrounding pixel (i, j). In the special case where $\Psi _{i,j}$=(i, j), the involution operator can be expressed as:

4 $$\begin{aligned} \hfil \mu _{i,j}=\Phi (X_{i,j})=W_1\sigma (W_0X_{i,j}). \end{aligned}$$

where $W_0\in {\mathbb {R}}^{\frac{C}{r} \times C}$, $W_1\in \mathbb {R}^{(K\times K\times G)\times \frac{C}{r} }$ and r represents the scaling factor, and $\sigma$ denotes the normalization and ReLU activation. The involution operation significantly reduces the numerical scale associated with $K^2$. As *K* increases, it does not lead to substantial changes in the number of parameters or computations. Therefore, when employing larger-sized involution operators to establish long-distance relationships between pixels, the resulting increase in parameters and computations remains modest and manageable within the model. The pseudocode of the involution operation can be expressed as follows:

**Algorithm 1:**
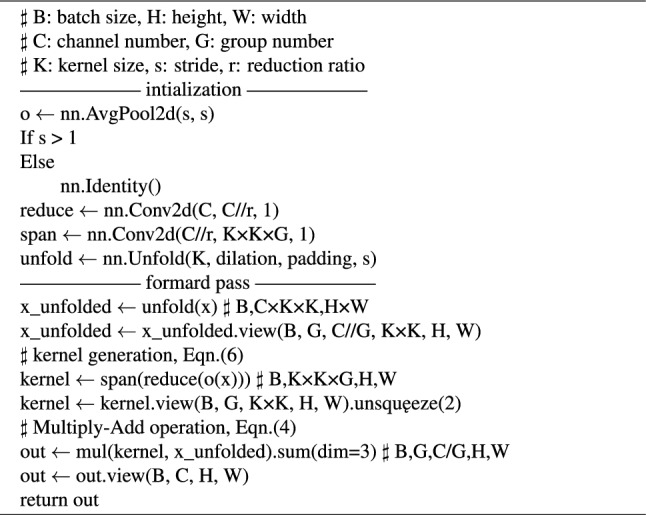
Involution in a PyTorch-like style

### Inference prediction relies on the utilization of multi-scale anchor boxes and multi-scale feature maps

During the inference prediction process on the input image, the category information predicted by each grid is multiplied by the prediction confidence of the target box to calculate the category confidence score for each target box. For the bounding box loss function, the IoU loss is employed^36^ to assess the dissimilarity between the predicted bounding box and the actual bounding box, and the constraint relationship between them can be expressed as:

5 $$\begin{aligned} \hfil Pre(Class_i|Object)\times Pre(Object)\times IoU\frac{truth}{pred}=Pre(Class_i)\times IoU\frac{truth}{pred}. \end{aligned}$$

In Eq. (5), the first term on the left side represents the predicted class probability for each grid, while the product of the second and third terms corresponds to the prediction confidence associated with each target box. This equation captures the likelihood that the predicted target bounding box belongs to a specific category, thereby deriving the category confidence score for each target bounding box. Subsequently, by establishing a threshold, target bounding boxes with scores below this threshold are filtered out. Following this filtering step, Non-Maximum Suppression (NMS)^37^ is applied to the remaining target bounding boxes to yield the ultimate detection result. In this process, each grid unit on the feature map at each scale is associated with three anchor boxes, each with distinct width and height ratios, serving as prior boxes. In addition to the border coordinates and width and height values, each anchor box also includes one confidence level and N category confidence levels. Consequently, the depth of each anchor box is 5+N. Our network utilizes three types (for a total of nine types) of anchor boxes to accommodate different scale features. These anchor boxes have specific sizes: 10$\times$13, 16$\times$30, 33$\times$23, 30$\times$61, 62$\times$45, 59$\times$119, 116$\times$90, 156$\times$198, and 373$\times$326. This diversity in anchor box sizes enables the network model to effectively detect targets of various sizes within the same image.

**Figure 5:**
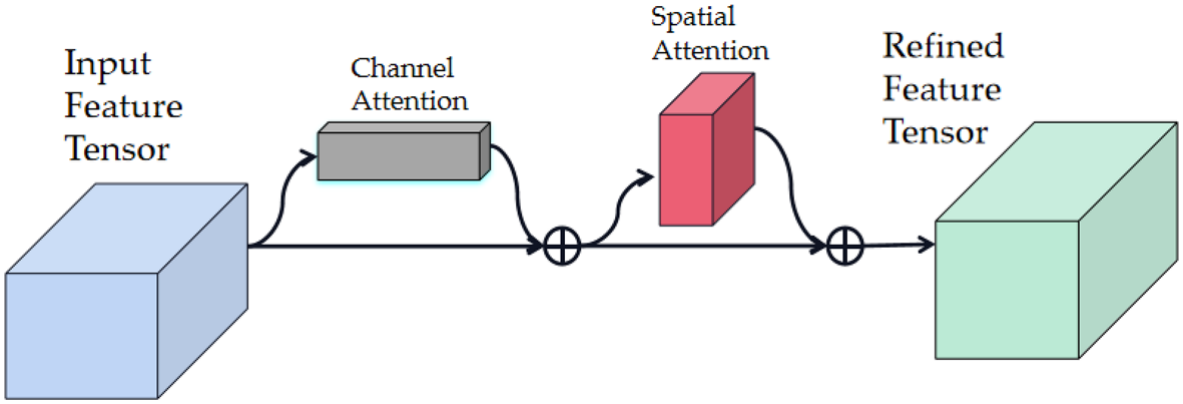
A dual-channel attention mechanism (CBAM).

From a structural perspective, the multi-scale target detection and output prediction for alligator gar images, based on the feature map generated by the network’s backbone, are accomplished within the pyramid-shaped multi-scale detection component of the proposed network model. In the first layer of this component (the 10th layer in the entire network), a dual-channel attention mechanism known as the Convolutional Block Attention Module(CBAM) is employed. This lightweight attention module empowers the network model to analyze the input feature map in both spatial and channel dimensions, with a specific focus on the target object to be detected. It combines the characteristics of spatial attention and channel attention, deriving attention weight coefficients through learning and reasoning, which are then applied to the feature map to enhance detection performance. The module’s structure is depicted in Fig. 5.

**Figure 6:**
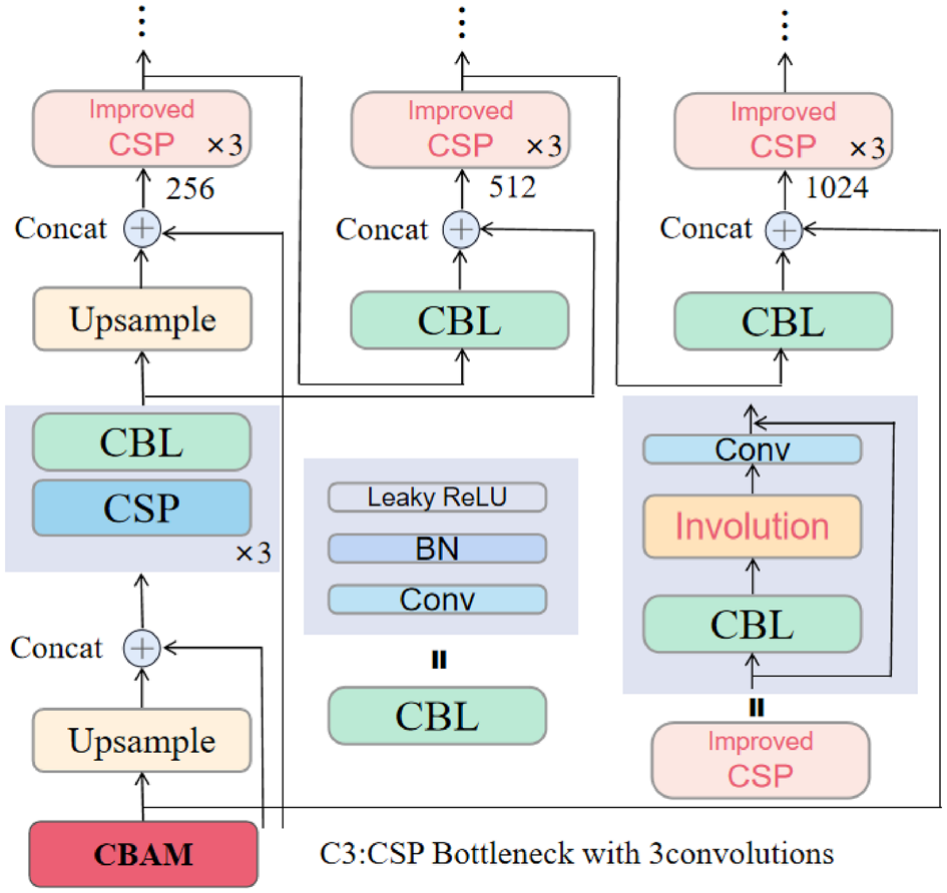
Illustrates the structure of the multi-scale detection and reasoning network. In this figure, CBAM is depicted as a dual-channel attention module, while Involution is represented as an inner convolution layer. The numerical values, such as 256 and 512, indicate the number of feature data channels in each respective layer.

Figure 5 illustrates that the Convolutional Block Attention Module (CBAM) primarily comprises two components: the channel attention module and the spatial attention module, which operate sequentially on input data. Initially, the CBAM module directs its attention to channels containing crucial information within the input feature map, generating attention-based feature information at the channel level. Subsequently, the output features are passed into the spatial attention module^38^, which prioritizes more significant spatial features, ultimately producing a weighted feature map. This refined feature map serves as the input data source for the network’s head, influencing related features in subsequent modules. Leveraging the positive impact of its attention mechanism, the network model can capture long-distance contextual information with fewer parameters, resulting in a more lightweight model. Notably, for the two multi-channel feature layers with 1024 and 512 (the actual number of output channels must be multiplied by the scaling factor), we opt for the involution method instead of convolution as the core filtering unit. This involution approach allows parameter sharing between channels, serving as an efficient feature extraction unit that captures comprehensive feature information while minimizing computational costs. The overall structure of the network’s head is depicted in Fig. 6.

### Lightweight feature extractor: FCInvNet

**Figure 7:**
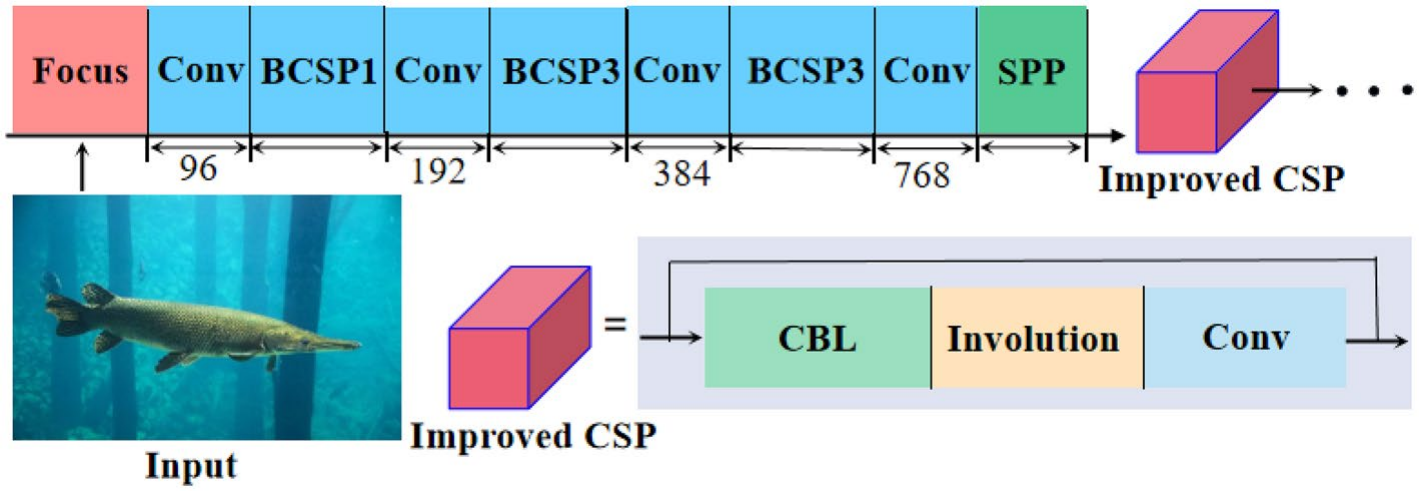
The feature extractor introduced in this study.

We constructed a lightweight feature extraction backbone network, denoted as FCInvNet, primarily through the incorporation of inner convolution layers. FCInvNet is characterized by its simplicity and effectiveness in feature extraction. Notably, when employed for the detection of images, such as those of the alligator gar, models utilizing this backbone network exhibit shorter inference times and reduced model capacities, all while achieving comparable accuracy to YOLOv5. The structural configuration of the backbone network is presented in Fig. 7.

The Focus structure slices the locally designated area of each channel in the input image and downsamples the original RGB input image to a 12-dimensional channel feature map with a 1/4 spatial size. Conv represents an ordinary convolution, while BCSP1 is a structural combination of Batch Normalization (BN) and CSPNet (Cross Stage Partial Network). The front-end convolutional kernel size of each CSPNet module is 3$\times$3, with a stride of 2, primarily performing downsampling operations. To mitigate the excessive computational cost resulting from duplicated gradient information during optimization, the CSP module initially divides the input feature information into two parts and subsequently concatenates them through a cross-stage hierarchy, effectively reducing the computational load while maintaining accuracy. CSPNet enhances the learning capability of Convolutional Neural Networks (CNN) while concurrently reducing computational complexity and memory consumption, contributing to a lightweight model.The SPP module employs 1$\times$1, 5$\times$5, 9$\times$9, and 13$\times$13-sized kernels to conduct maximum pooling operations on the input features. It then concatenates the feature maps from various scales. Utilizing SPP provides a broader range of feature sensing compared to using only k$\times$k maximum pooling. The final module, CSPNet, introduces the inner convolution module. During feature extraction, it not only generates more effective feature map patterns but also efficiently reduces numerous redundant calculations among high-dimensional channels. This approach maintains conciseness and detail. For further details, please refer to the “Lightweight feature extractor: FCInvNet” section of this article.

Figure 7 reveals that the final output layer employs the involution method as the feature extraction unit. Given the substantial number of output feature map channels in this context, experimental measurements have demonstrated that utilizing convolutional layers would lead to the generation of 4.1 million parameters. In contrast, employing the involution method for processing involves only 1.8 million parameters in the calculations. This article astutely capitalizes on the involution operators’ ability to share parameters across channels. It utilizes a feature processing module with involution at its core to circumvent the challenges posed by convolutional layers’ inability to share parameters among channels when dealing with large-scale channels, thereby mitigating redundant computations.

Compared to YOLOv5, which comprises Focus^39^ and CSPDarknet53^40^ (hereinafter referred to as F-CSPDarkNet53), our model exhibits a marked reduction in computational complexity, a slight enhancement in accuracy, and significantly improved detection efficiency when compared to the ResNet^41^ and DarkNet^42^ series. This renders it better suited for the task requirements of real-time alligator gar detection. Table 1 presents quantitative results based on the alligator gar image dataset constructed in this study as the experimental subject.

**Table 1:** Comparative quantitative results between the feature extractor proposed in this article and YOLOv5with F-CDarkNet53 as the backbone network.

Backbone	$AP_{50}$	$AP_{75}$	Para Mnt (M)	GFLOPs	FPS
FCDarkNet53	96.6	57.8	12.4	50.6	13.66
FCInvNet (ours)	97.2	60.3	10.1	48.6	21.93

Average accuracy measured under two IoU thresholds (AP50 and AP75). Para Mnt represents the number of parameters involved in the operation, GFLOPs denotes the floating-point operations per second, measured in GB, and FPS indicates the detection speed.

The quantitative results presented in Table 1 were obtained under identical environmental settings. We conducted 100 rounds of training and learning on the alligator gar dataset using an RTX3060 (12G) graphics card. As shown in Table 1, the feature extraction module in our approach notably improved detection speed. Specifically, when using our method’s feature extractor (with the remaining structures equivalent to YOLOv5), the generated model achieved a detection speed 1.61 times faster than YOLOv5.

### Loss function

We employ the identical loss function as YOLOv5, comprising Classification loss, Localization loss, and Confidence loss, as delineated in formula (6). Binary cross-entropy is utilized for both confidence and classification loss, whereas the positioning loss is computed using the *CIOU* Loss. Formulas (7) and (8) succinctly represent the category loss function and confidence loss function.

6 $$\begin{aligned} \hfil Loss= & {} Loss_{class}+Loss_{loc}+Loss_{conf}. \end{aligned}$$

7 $$\begin{aligned} \hfil Loss_{class}= & {} -\sum _{i=0}^{K\times K} I_{ij}^{obj}\sum _{c\in classes}\left[ \hat{P}_{i}^{j}log(P_{i}^{j})+(1-\hat{P}_{i}^{j})log(1-P_{i}^{j}) \right] . \end{aligned}$$

8 $$\begin{aligned} \hfil Loss_{conf}= & {} -\sum _{i=0}^{K\times K} I_{ij}^{obj}\left[ \hat{C}_i^jlog(C_i^j)+(1-\hat{C}_i^j))log(1-C_i^j) \right] \nonumber \\{} & {} -\lnot \alpha _{obj}\sum _{i=0}^{K\times K}\sum _{j=0}^{N}\lnot I_{ij}^{obj}\left[ \hat{C}_i^jlog(C_i^j)+(1-\hat{C}_i^j))log(1-C_i^j) \right] . \end{aligned}$$

Here, $K\times K$ is the number of grids into which the input image is divided, while *N* signifies the number of anchor boxes corresponding to each grid. Furthermore, $I_{ij}^{obj}$ denotes the anchor box containing the target, $\lnot I_{ij}^{obj}$ represents the anchor box devoid of the target, and $\lnot \alpha _{obj}$ symbolizes the confidence loss weight of the anchor box that remains ineffective. Our focus now shifts towards elucidating the bounding box loss, commonly referred to as Localization loss.

The initial bounding box loss employs *IoU* loss to gauge the intersection ratio of the actual box and the predicted box, represented as (9). However, it is worth noting that when the actual box and the predicted box fail to overlap, the *IoU* value becomes 0. In such instances, the gradient becomes non-informative, and optimization issues may arise due to the absence of feedback about the spatial relationship between the two boxes.

9 $$\begin{aligned} \hfil IoU=\frac{|A\cap B|}{|A\cup B|}. \end{aligned}$$

**Figure 8:**
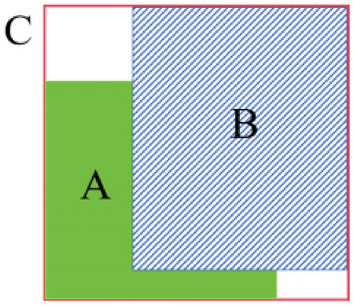
Illustrates the schematic diagram of the minimum bounding box in GIoU loss.

The *GIoU* (Generalized Intersection over Union) loss^43^ effectively addresses the scenario in which the *IoU* value equals 0 by introducing the concept of a minimum bounding box. Consider *A* and *B* as representations of the actual target box and the predicted box, as illustrated in Fig. 8.

The *GIoU* loss can be expressed as formula (10), where the minimum bounding box *C* encompasses both the green box *A* and the blue shaded box *B* along their two sides.

10 $$\begin{aligned} \hfil GIoU=IoU-\frac{|C\backslash (A\cup B)|}{C}. \end{aligned}$$

**Figure 9:**
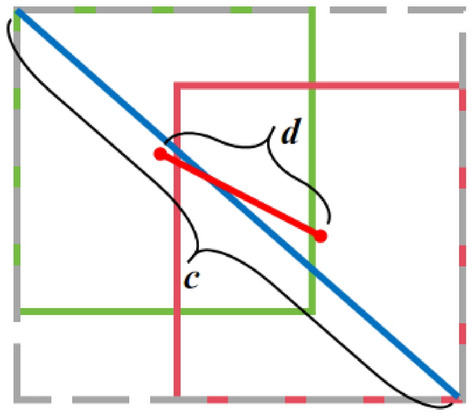
Schematic diagram of *DIoU* Loss principle.

However, when the predicted box and the real box are entirely contained within each other, *GIoU* will degrade into *IoU*. On the other hand, when they intersect, convergence occurs at a slower rate along both the horizontal and vertical directions. The *DIoU*^44^ loss introduces the prediction box and the real box, taking into account the distance between their center points and their diagonals, as illustrated in Fig. 9.

In this context, *C* represents the diagonal of the minimum bounding box, while *D* signifies the distance between the center points of the true and predicted boxes. The *DIoU* loss function is mathematically expressed as follows:

11 $$\begin{aligned} \hfil L_{DIoU}=1-IoU+\frac{\rho ^2(b,b^{gt})}{c^2}. \end{aligned}$$

$\rho ^2()$ represents the Euclidean distance, *b* and $b^{gt}$ represent the center points of the predicted box and the true box, and c represents the diagonal of the minimum bounding box of the two boxes. In cases where the predicted box and the real box fully contain each other, the distance between their center points becomes a crucial reference, offering guidance for further optimization. It also addresses the issue of slow convergence along the horizontal and vertical directions when the two boxes intersect. The *CIoU*^45^ loss introduces the aspect ratio to assess the consistency between the width and height of the predicted box and the true box, which can be expressed as follows:

12 $$\begin{aligned} \hfil L_{CIoU}=1-IoU+\frac{\rho ^2(b,b^{gt})}{c^2} +\alpha v. \end{aligned}$$

Among these terms, $\alpha v$ represents the aspect ratio component introduced in the *CIoU* loss. In contrast to *IoU*, *GIoU*, and *DIoU*, *CIoU* not only converges more rapidly but also incorporates considerations of the overlapping area, center-point distance, and aspect ratio, thereby resulting in a more precise regression frame. In this article, we utilize the *CIoU* loss function for bounding box regression.

## Experiment results

### Alligator gar self-collected data set

**Figure 10:**
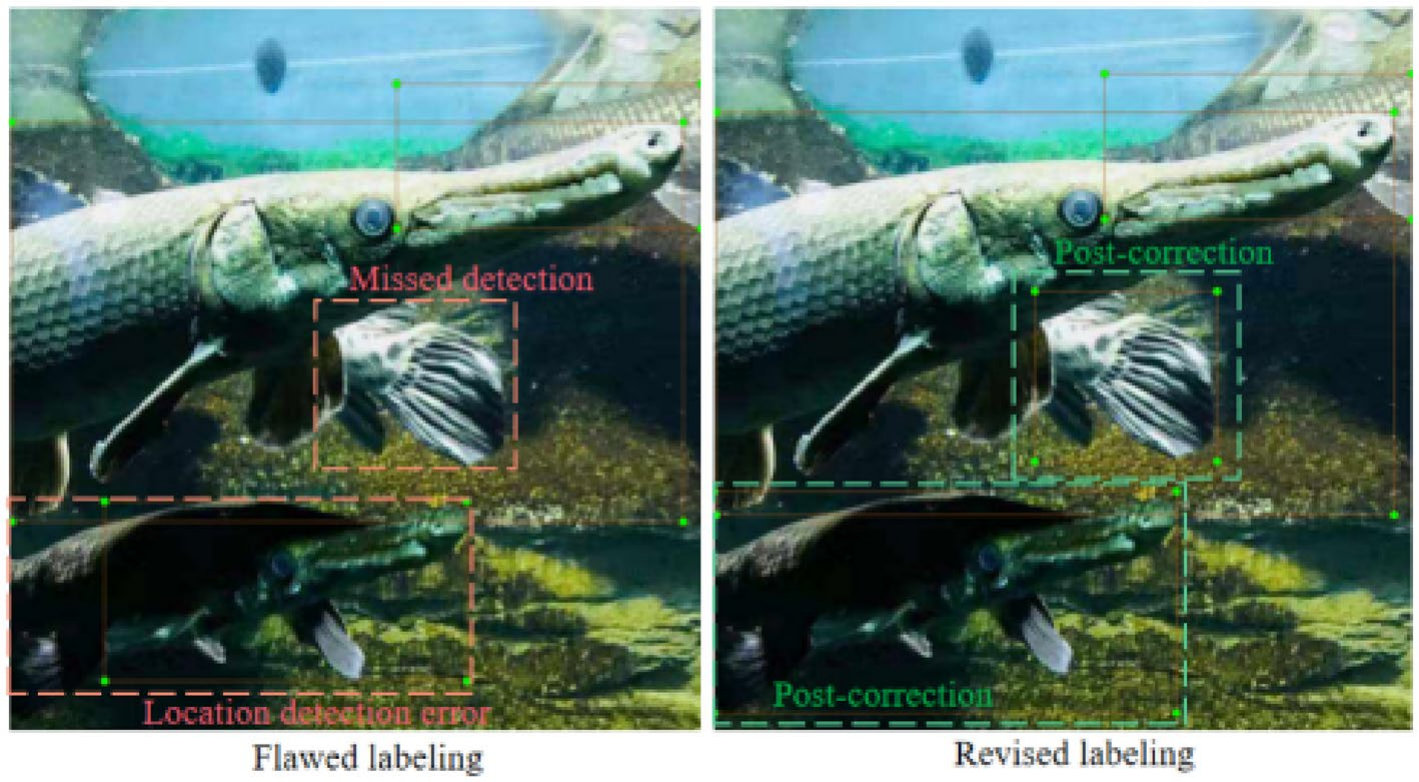
Examples before and after manual review and proofreading of samples.

We conducted a comprehensive data collection effort, acquiring a diverse array of alligator gar images. These images were captured under varying shooting angles, within a spectrum of water environments, and under distinct lighting conditions. The dataset encompasses different growth stages of alligator gar, featuring both complete and partially visible specimens. In total, our dataset comprises a substantial collection of 1400 high-resolution alligator gar images. These images have been meticulously and manually annotated following the format of the VOC dataset^46^, utilizing the annotation tool known as “labelimg”. Notably, each image has been assigned a single classification label, denoted as “Atra-spa”. Rigorous quality control procedures were implemented to ensure the accuracy and reliability of the annotated data, as exemplified in Fig. 10.

The dataset has been systematically partitioned into three distinct subsets to facilitate the development and evaluation of machine learning models. These subsets are as follows:

- Training Set: This segment comprises 840 images, serving as the foundational dataset for training and fine-tuning machine learning models.
- Validation Set: The validation set consists of 280 images. It is intended for model validation and parameter tuning, ensuring the model’s robustness and generalization.
- Test Set: Comprising an additional 280 images, the test set is reserved for rigorous evaluation and performance assessment of the trained models.

Figure 10 provides a visual representation of specific samples from these delineated subsets, offering insight into the diversity and distribution of images across the training, validation, and test sets.

**Figure 11:**
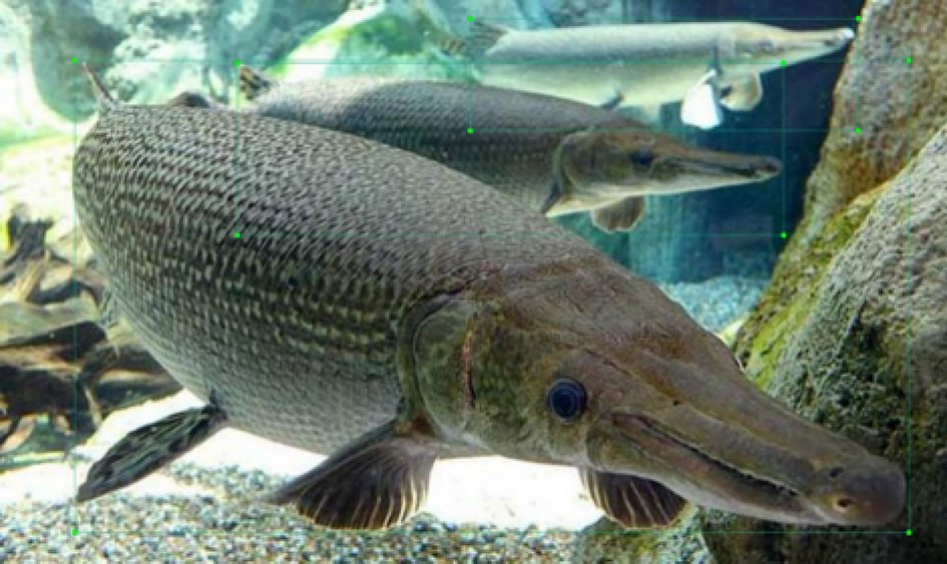
A sample derived from our alligator gar target detection dataset.

The insights provided by Fig. 11 underscore the distinctive challenges inherent to alligator gar target detection within aquatic environments. These challenges are as follows:

- Multiple Alligator Gars Swimming Together: Instances where multiple alligator gars coexist in proximity introduce complexities related to distinguishing individual targets within a cluster.
- Blurring of Distant Alligator Gars Due to Refraction: Distant alligator gars may appear blurred or distorted as a consequence of refraction effects caused by underwater conditions, thereby impeding accurate detection.
- Similarity Between Alligator Gar and Surrounding Objects: The visual similarity between alligator gars and their immediate surroundings poses difficulties in discerning between the target and non-target elements, demanding more nuanced feature extraction.
- Challenging Angles: The angular orientation of alligator gars relative to the monitoring lens can further exacerbate the detection challenge, as it may obscure crucial identifying characteristics. These complex scenarios variably impact both the speed and accuracy of alligator gar detection, necessitating the development of sophisticated algorithms and models capable of addressing these intricacies within real-time monitoring applications.

**Figure 12:**
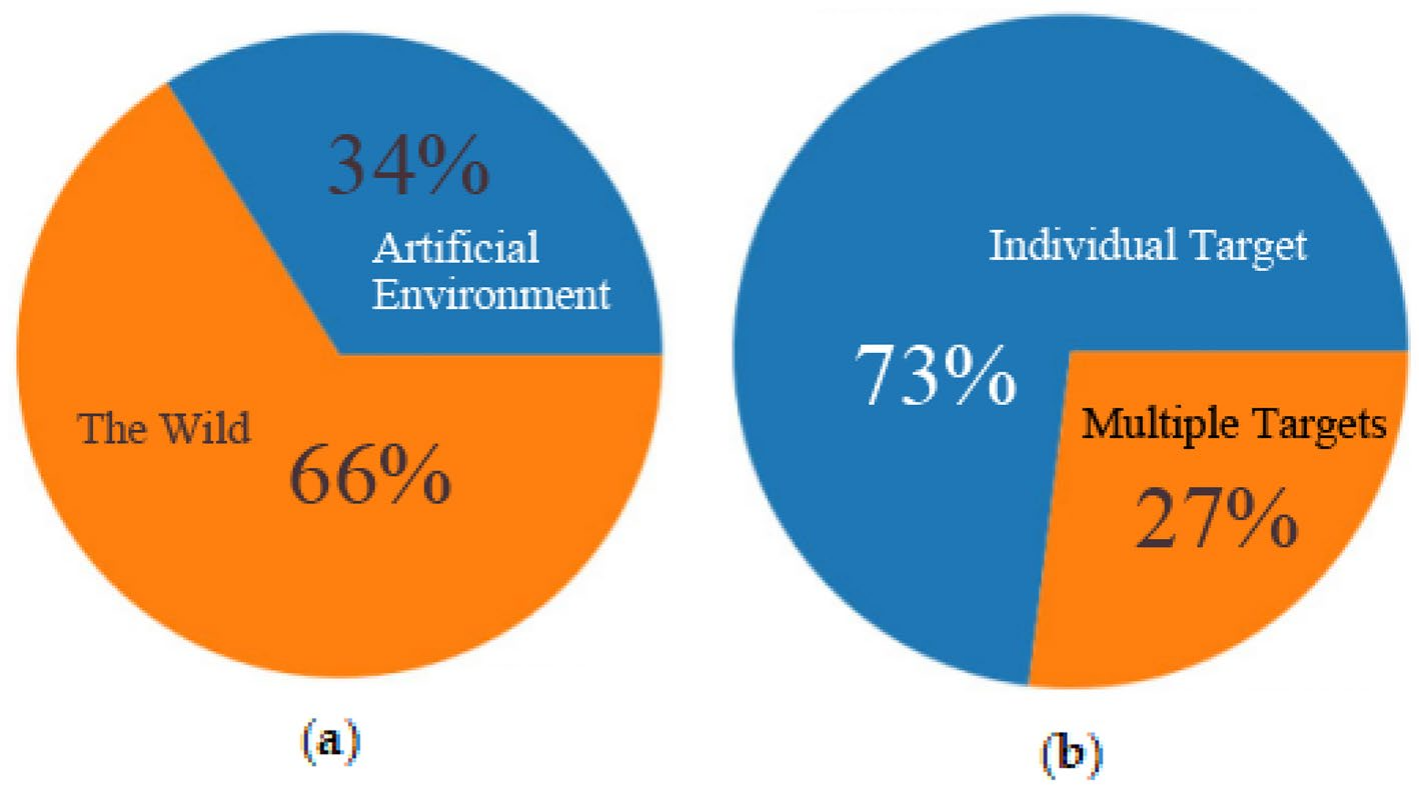
Schematic diagram of the composition of the alligator gar data set. offering a comprehensive overview of its structural characteristics: (**a**) Categorized based on living environments: Approximately 34% of the images depict scenes from artificial breeding environments, while the remaining 66% capture images from natural, wild settings. (**b**) Classified according to the quantity of alligator gar present in each image: The distribution reveals that 73% of the images feature a solitary alligator gar, whereas 27% showcase multiple individuals of the species.

The composition of our dataset is delineated in Fig. 12, This dataset composition ensures a well-rounded representation of various real-world conditions, facilitating the development and evaluation of alligator gar detection models that are robust and adaptable across a spectrum of environments and scenarios.

### Training

The experimental machine configuration employed in our study is as follows: the CPU used is a 12th Gen Intel(R) Core(TM) i7-12700F, with 16GB of RAM, and the graphics card is an NVIDIA RTX3060 with 12GB of VRAM. We utilized a parallel computing environment with CUDA 11.6, Python version 3.7, and PyTorch version 1.9.1+cu111. The operating system installed on the machine is Windows 11.

For our training process, we set the following specific parameter values:

- Learning rate: 0.001.
- Momentum: 0.6.
- Batch size: 16.
- Optimizer: Adam.

We conducted training on the alligator gar dataset for a total of 100 iterations. Additionally, we performed training on the VOC public dataset for a total of 300 iterations.

**Figure 13:**
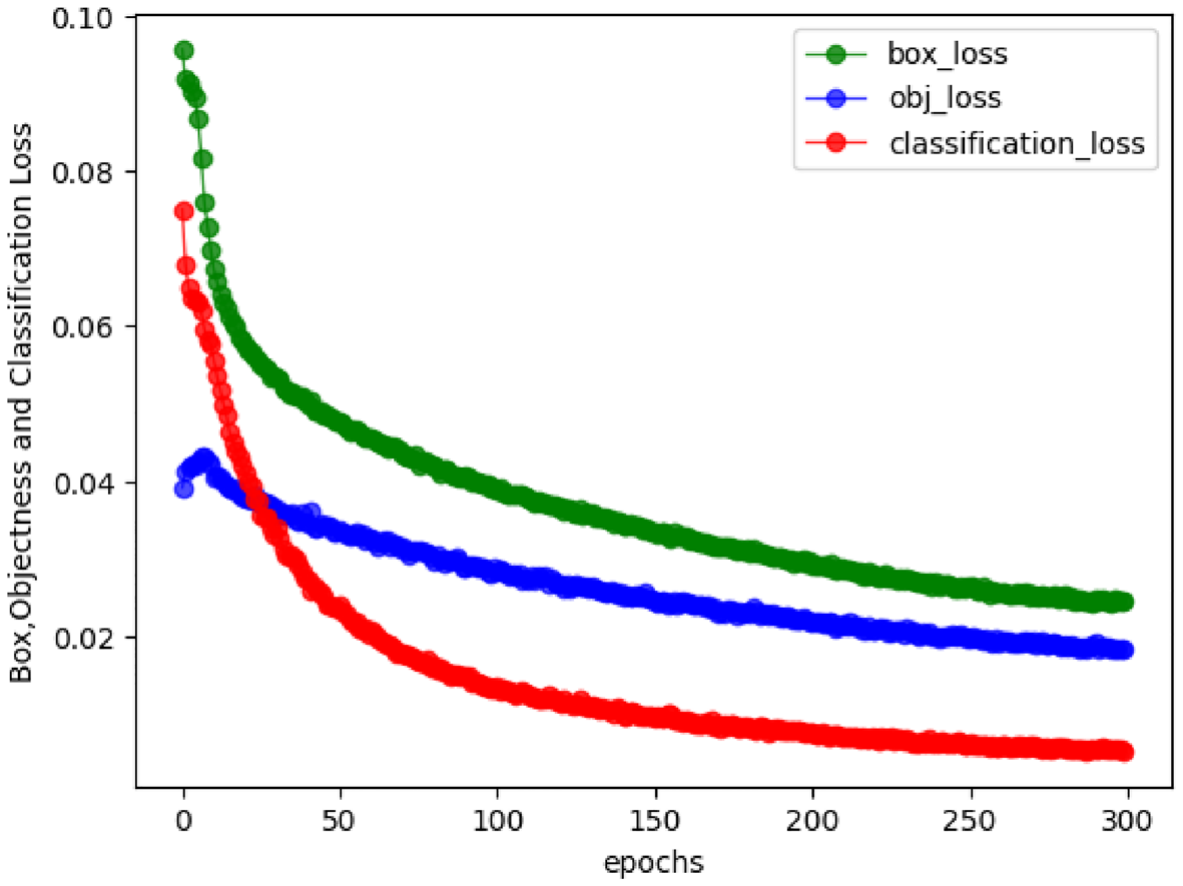
Illustrates the trend of mean values for several loss functions over the course of 300 rounds of training using the method applied to the PASCAL VOC dataset.

We initially utilized the custom data set of Alligator Gar for training and validating both this article and the associated network models. Following the attainment of favorable experimental results, we extended our training and validation process to incorporate the VOC2007 dataset, along with a portion of the VOC2012 public data sets, with the objective of assessing the method’s generalization capability. During the training process of the custom Alligator Gar dataset, we took steps to ensure the model’s adaptability to diverse water environments and varying lighting conditions. To enhance the model’s robustness, we augmented the dataset by introducing an additional 1,400 images. Specifically, within the Alligator Gar dataset, we randomly selected 400 images for low-light processing and duplicated them. Additionally, we randomly selected and duplicated 240 images to introduce noise, thereby expanding the dataset and increasing the diversity of samples. While training on the PASCAL VOC2012 public dataset, we employed the original data samples without making any alterations for the training process. For detailed insights into the training process, including the mean iteration trend of the loss function related to target box IoU feedback, target reasoning, and classification prediction, please refer to Fig. 13.

Figure 13 provides a clear insight into the training process, showing a consistent decrease in the prediction frame, classification recognition, and confidence loss throughout. Notably, the classification loss exhibited a gradual and smooth decline, starting from nearly 0.08 initially and eventually falling to less than 0.01. Taken together, these results indicate a relatively favorable training outcome. It is worth noting that all the training and verification procedures detailed in this article were carried out within the PyTorch framework.

### The actual detection results obtained in the alligator gar target detection task

**Figure 14:**
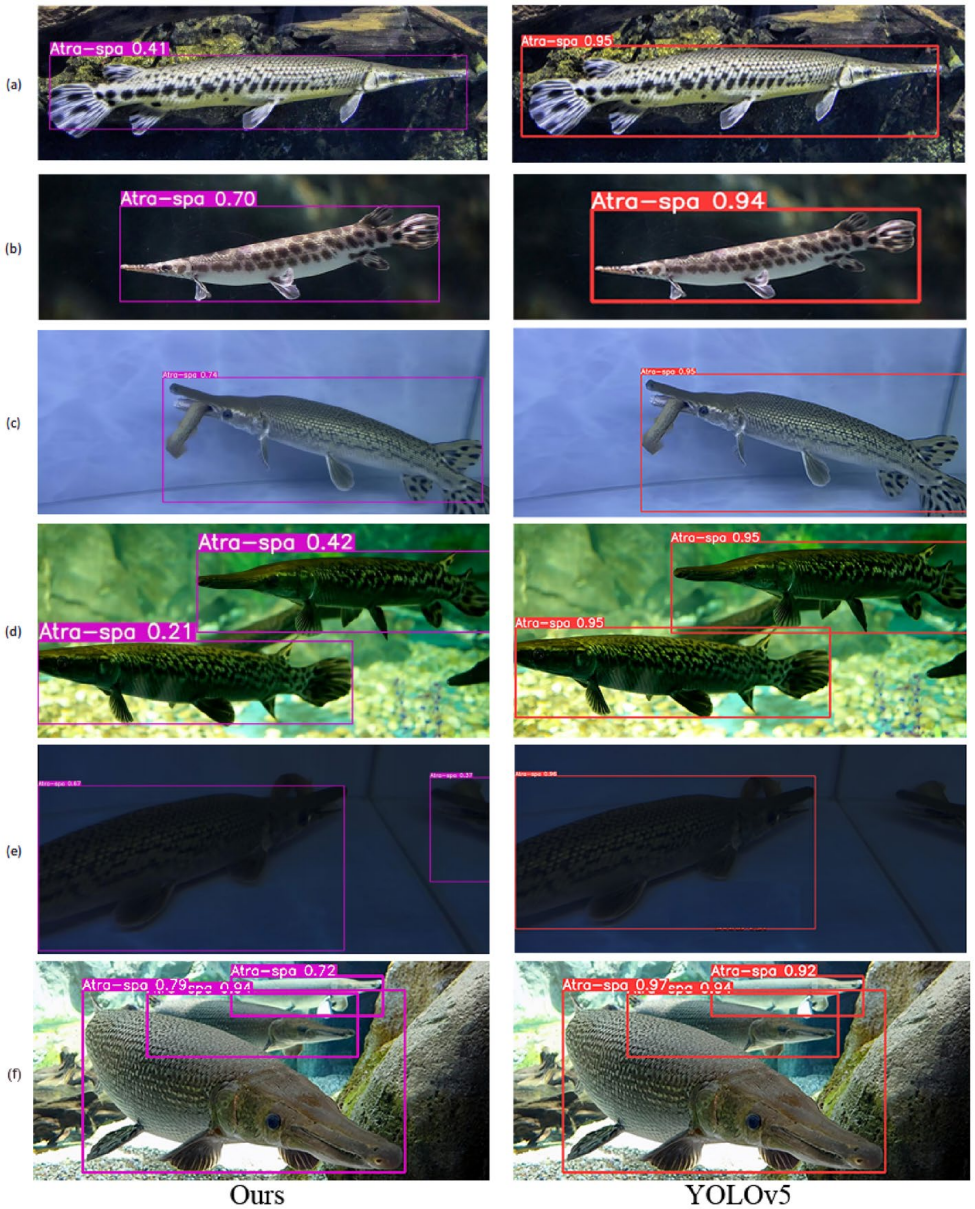
Provides an example illustrating the effectiveness of the method described in this article for detecting Alligator Gar targets. On the left side are the detection results of this method, while on the right side is the inference effect of YOLOv5.

In the practical detection of image samples within the Alligator Gar test set, our method has demonstrated strong performance. Despite the variability in water quality within the dataset and potential challenges like low light conditions, image deformations, blurriness due to refraction, and complex environmental backgrounds, our model has consistently performed well. It is noteworthy that some background content may share visual similarities with the detected target; however, our model remains effective in accurately detecting Alligator Gar targets. The results affirm that the approach presented in this paper effectively avoids issues such as overfitting^47^, underfitting^48^, gradient disappearance, and gradient explosion^49^. For specific details and visual representations of the detection results, please refer to Fig. 14.

**Figure 15:**
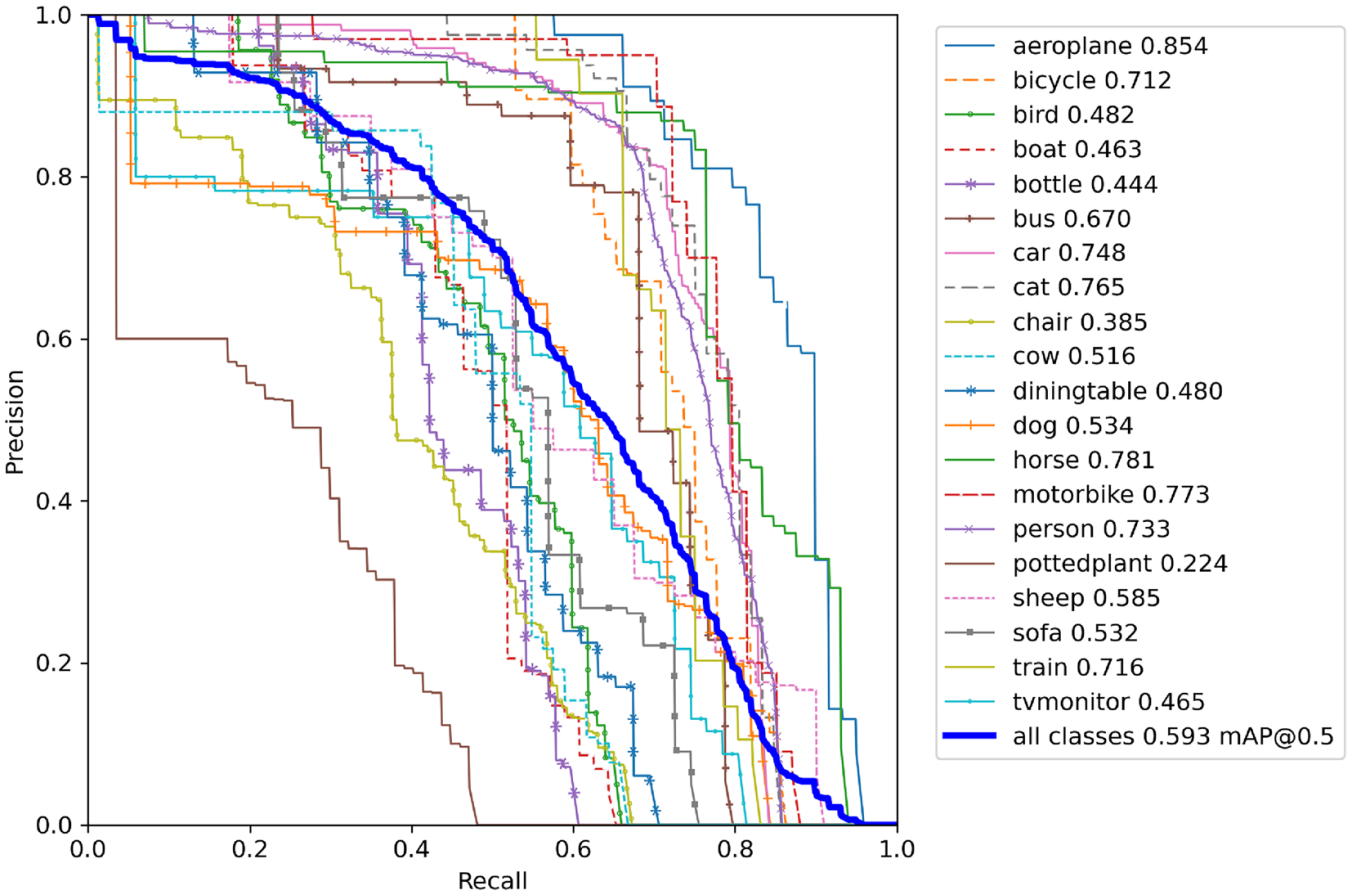
Displays the Precision–Recall (P–R) curve resulting from 300 rounds of iterative training of the method on the PASCAL VOC dataset.

- As depicted in Fig. 14a, it is evident that despite the complexity of the environmental background in the image and the presence of surrounding objects with somewhat similar surface textures to the Alligator Gar target, the method outlined in this paper successfully detects the target object accurately. This method excels in position fitting.
- Moving on to Fig. 14b, even though the background containing the rear 1/3 of the Alligator Gar’s body shares certain similarities in color information with the target itself, our method effectively identifies the Alligator Gar, marking its location accurately.
- In Fig. 14c, despite a slight deviation in target positioning, the detection remains acceptable, particularly considering that the Alligator Gar is in a feeding state, and the overall image is relatively dark.
- In Fig. 14d, the low illumination of the entire image affects the texture features of the foreground Alligator Gar to some extent. Nevertheless, our method accurately identifies and detects multiple targets.
- Figure 15e presents a significant challenge with very low illumination, but even in such conditions, our method demonstrates its ability to identify and detect multiple targets with relative accuracy. YOLOv5 failed to detect the section of the alligator gar on the right side of the image.
- Figure 15f, the detection effect of this method is comparable to that of YOLOv5.

In summary, the method proposed in this article exhibits strong robustness in the Alligator Gar detection task.

### Quantitative results analysis

The experimental quantitative results indicate that the method proposed in this paper deserves recognition. To expedite the assessment of the method’s advancement, we initially conducted experiments on a small-scale dataset of Alligator Gar images. Our method outperformed the one-stage target detection algorithms YOLOv3 and SSD513 across various performance indicators. Notably, the detection accuracy of our method slightly surpassed YOLOv5 (medium), and, additionally, our model’s inference time was 35.1% faster than YOLOv5 (medium), while the model size was only 86.1% of YOLOv5 (medium). In comparison to the state-of-the-art (SOTA) method YOLOv8 (medium), our approach exhibits an inference time that is 71.8% faster, and the model size is also 71.4% of YOLOv8 (medium). For detailed metrics and comparisons, please consult Table 2.

**Table 2:** Experimental results on the alligator gar dataset.

Method	Backbone	$AP_{50}$	$AP_{75}$	FLOPs (G)	FPS	Model size (m)
Faster RCNN-FPN^50^	ResNet101-FPN	93.0	54.6	–	7.5	315.7
SSD513^51^	ResNet101	88.7	47.1	–	10.5	113.5
DETR^52^	ResNet101	94.7	55.1	89.3	8.1	161.2
YOLOv3	DarkNet53	92.6	49.5	153.2	22.4	123.3
YOLOv5	FCDarkNet53	96.6	57.8	50.6	33.3	39.7
YOLOv8	FCDarkNet53	97.6	59.3	82.9	26.2	47.9
Ours	FCInvNet	97.0	58.1	44.7	45.0	34.2

Table 2 clearly highlights the primary advantage of our method, which lies in its superior inference speed. By harnessing the power of learning from enhanced input data features, our method exhibits heightened recognition capabilities, particularly when dealing with Alligator Gar image data characterized by low illumination and increased noise. This enhanced capability translates into improved robustness and adaptability, making our method well-suited for the complexities of actual Alligator Gar imaging target detection tasks, including scenarios where light is refracted in water and environmental factors such as turbid water quality influence image illumination. To further evaluate the random image detection capabilities of our method for common applications, we conducted experiments using public datasets to validate its effectiveness. Our method was employed to perform target detection experiments on images from the relatively complex PASCAL VOC 2007 and a portion of the VOC 2012 datasets. Both datasets encompass 20 distinct classes of objects, enabling a comprehensive assessment of our method’s performance in diverse and complex scenes. For detailed results of the detection of various object targets, please refer to Table 3.

**Table 3:** Experimental results on Pascal VOC dataset.

Method	Backbone	$AP_{50}$	$AP_{75}$	FLOPs (G)	FPS	Model size (m)
Faster RCNN-FPN	ResNet101-FPN	58.6	32.1	–	16.0	316
SSD513	ResNet101	55.9	27.7	–	22.5	115
DETR	ResNet101	60.1	32.6	90.4	17.3	165.5
YOLOv3	DarkNet53	57.4	29.3	155.2	53.9	123.6
YOLOv5	FCDarkNet53	60.1	34.2	50.6	73.0	42.6
YOLOv8	FCDarkNet53	60.4	35.1	83	57.5	49.5
Ours	FCInvNet	59.3	35.1	44.7	108.3	31.0

As indicated in Table 3, the method presented in this paper outperforms other algorithms across many metrics when considering mAP@0.5. This superiority is primarily attributed to the efficient characteristics of the involution operator. When applied to the detection of PASCAL VOC images, our method achieves an impressive speed of 108.3 frames per second, while YOLOv5 operates at 73 frames per second. Consequently, the detection efficiency of our method surpasses that of YOLOv5 by 48.4%, the inference speed is 88.3% faster than YOLOv8. Furthermore, our approach manages to reduce the FLOPS and parameter count, resulting in a more compact model. It is crucial to note that while the human visual system captures continuous images at a rate of 60 frames per second (fps), the FPS indicator may inevitably vary when processing large or even ultra-large-resolution input images in diverse applications. In such cases, there may be a reduction in FPS. Algorithm models boasting higher FPS levels, as validated through public datasets, may still prove effective in certain scenarios. Based on the quantitative results presented in Tables 2 and 3, the exceptional performance of this method stems from its utilization of involution, a novel technique, within the feature layer responsible for generating large-scale channels in the network structure. The feature processing unit not only adeptly captures diverse feature information through flexible and effective grouping but also significantly mitigates resource consumption arising from redundant calculations within the network model. Strategically deploying concise involution modules at appropriate locations within the network renders the model lighter and more adept at discerning feature information, thereby achieving heightened efficiency with minimal effort.

As indicated in Tables 2 and 3, particularly for the dynamic target of the Alligator Gar Mountain, while the accuracy of this method may exhibit slight variations compared to various algorithms, our primary objective is to achieve swift detection of the Alligator Gar target. Additionally, it is essential to deploy the generated model on resource-constrained terminal devices. Therefore, the focus of this article is consistently on making the model lightweight. For any disparities in accuracy, we will opt for a more suitable, noticeably lightweight model that can rapidly accomplish dynamic target detection, aligning with our overarching objectives. Based on the quantitative results in Tables 2 and 3, the method proposed in this article emerges as the most fitting choice.

Upon comparing the aforementioned two experiments, it becomes evident that in the context of multi-category detection tasks, the utilization of an efficient and concise involution operator may, to some extent, result in a reduction of detection accuracy. However, when weighed against the enhanced detection efficiency and the substantial reduction in the output model’s size, the trade-off is deemed worthwhile. Furthermore, the shared parameters within channel groups and the distinct parameters in the spatial dimension, based on involution, facilitate the perception of characteristics in different local areas. While there might be a temporary loss in accuracy, this tends to rebound with an increase in the number of training iterations, a phenomenon that will be systematically observed and reflected upon in subsequent ablation experiments.

The network model we developed incorporates modules with the involution operator at the core of both the backbone and the detection output sections. Additionally, it employs a dual-channel attention mechanism in the detection part. These components fulfill distinct roles, contributing to the method’s enhanced performance. For a graphical representation of the PR curve (Precision-Recall Curve), please refer to Fig. 15.

### Ablation experiment analysis

To assess the specific advantages offered by these key modules, relevant ablation experiments were conducted. These experiments were carried out while utilizing the multi-scale detection head built on the foundation of convolution layers. The results of these experiments are summarized in Table 4.

It appears that the table is meant to demonstrate the impact of incorporating the involution module at different layers within the multi-scale detection part of the network. The ablation experiments likely involve assessing the model’s performance with various configurations, specifically:

- FCInvNet: The backbone network constructed in this paper, responsible for extracting image features. H23C3Inv: The utilization of the involution module at the last layer of the multi-scale detection part, which corresponds to layer 23 in the network.
- H20C3Inv: The inclusion of the involution module at layer 20 of the multi-scale detection part. It appears that “CBAM10” refers to the inclusion of a dual-channel attention mechanism in the first layer of the multi-scale detection part, specifically at layer 10.

To comprehensively assess the specific contributions of the involution module, CBAM, and other components in the current network model, we conducted 10 repeated ablation experiments. Subsequently, we computed the averages of the obtained quantitative indicators, leading to the following experimental results:

**Table 4:** Systematic experimental results on the Pascal VOC Dataset.

Method	$AP_{50}$	$AP_{75}$	FLOPs (G)	FPS	Model size (m)
FCInvNet	60.7	33.7	48.9	87.3	38.1
FCInvNet +H23C3Inv	61.9	34.2	47.1	95.2	33.6
FCInvNet+H23C3Inv+H20C3Inv	62.4	35.1	45.3	101.8	32.5
FCInvNet+H23C3Inv+H20C3Inv+CBAM10(ARD-Net)	61.5	34.3	44.7	109.2	31.0

The results presented in Table 4 indicate that when continuing to introduce the involution module at layer 23, based on the backbone network that originally contained an involution module, there was a slight drop in AP_50_ for the overall network. However, there was a significant increase in FPS by 7.9%, along with a reduction in FLOPS by 1.8G and a decrease in generated model size by 12%. Subsequently, by further incorporating and using the involution module in the 23rd layer, the network’s AP50 and FPS increased by 0.8% and 6.9%, respectively, while FLOPS decreased by 3.8%, and the generated model size decreased by 3.4%. These outcomes can be attributed to the fact that the involution mechanism effectively shares parameters between channels, particularly in the 20th and 23rd layers of the network in this paper, which contain a substantial number of channels. This parameter sharing reduces computational overhead and resource consumption. Additionally, the CBAM module, which combines spatial attention and channel attention, requires fewer parameters to gather long-range information for feature maps directly imported from the backbone network, resulting in a more efficient and compact model. This approach optimizes both computational efficiency and model portability.

**Figure 16:**
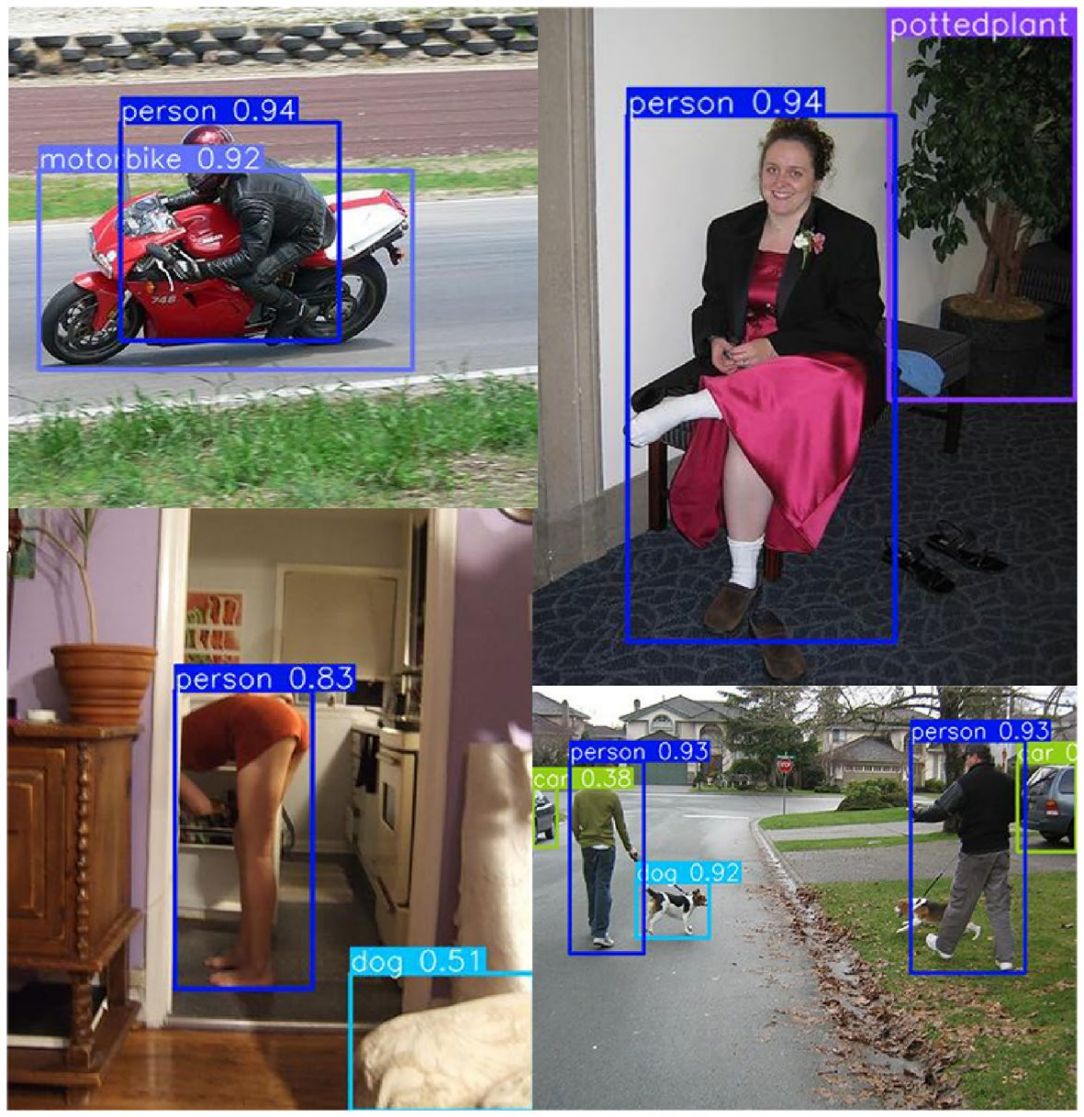
Examples of test results of this method on the PASCAL VOC dataset.

Analysis of the experimental results presented in Table 4 reveals that the utilization of the involution module, whether employed or not, exhibits no direct correlation with the Average Precision (AP). However, it is intricately linked to the specific location of the involution module and demonstrates a certain relationship with the random initialization of parameters. For examples of object detection in typical scenes using this method, please consult Fig. 16.

### Not applicable

The introduction of the involution module with the involution operator at its core provides enhanced capabilities to reflect spatial information in multi-channel feature maps during image feature extraction. This not only ensures the accuracy of image feature extraction but also leverages parameter sharing between channels, leading to a significant reduction in model cost. This reduction in computational complexity and operational consumption results in a more lightweight pre-trained model.

However, it’s important to note that replacing all convolution modules with involution modules might not always be the optimal strategy. To verify this, you conducted experiments, and I assume you assessed the performance of models when different combinations of convolution and involution modules were used. The results likely indicated that a balanced mix of convolution and involution modules, depending on the specific task and dataset, could provide the best trade-off between computational efficiency and model performance. The experiments help identify the right balance in utilizing these modules to achieve the desired outcomes.

The experiment involved replacing layer 6, which corresponds to the second BSP3 module in the backbone network presented in this paper, with an involution module. After 300 rounds of iterative training, several observations were made:

- The number of model parameters was significantly reduced.
- However, the training time increased.
- The AP50 index of the model dropped to 32.2.

In continuation of the experiment, after replacing the fourth layer, which corresponds to the first BSP3 module in the backbone network, with an involution module following the replacement described in 5.1, several results were observed:

The AP50 of the overall model dropped significantly to 14.3.

Maintaining the unchanged backbone network while replacing all pre-output parts of the detection head with involution modules resulted in the following observations:

- A decrease in the mAP of the model.
- An extension of the training time.
- A greater demand for GPU resources.

The specific experiments conducted have allowed us to draw several conclusions. The adverse results observed can be directly attributed to the use of involution modules. While the involution operator has the advantage of enabling multi-channel feature data to share parameters between channels, significantly reducing the number of model parameters, it has certain limitations.

Each feature map channel typically contains essential information related to different aspects or modes of input feature map data. When parameters are shared between channels, especially in layers with a large number of channels, each group of channels shares the same set of parameters. This sharing can result in some loss of pattern information. However, for larger-scale feature information, the benefits of parameter reduction tend to outweigh the losses.

On the other hand, in layers with a smaller number of channels, the opposite effect occurs. Sharing parameters leads to more significant losses than savings. In the case of shallower layers, this approach can significantly reduce the diversity of channel information, ultimately resulting in a decrease in mAP, which negatively impacts model performance.

This analysis highlights the importance of carefully considering the application of involution modules and recognizing that the effectiveness of parameter sharing can vary depending on the layer’s characteristics. A one-size-fits-all approach may not be optimal, and thoughtful selection of layers for the inclusion of involution modules is necessary to maintain or enhance model performance.

Through experimentation, we discovered that employing involution enhances the performance of the network model when the number of channels exceeds 512. Moreover, the performance improvement is more significant with a larger number of channels. Conversely, when the network layer comprises fewer than 512 channels, utilizing involution leads to a degradation in the network model’s performance, with a more pronounced decline observed with fewer channels. Please refer to Table 5 for further details:

**Table 5:** Experimental quantitative results on the Pascal VOC Dataset.

Backbone	$AP_{50}$
No.2 layer (64 channels)	8.9
No.4 layer (128 channels)	13.7
No.6 layer (256 channels)	18.5
No.8 layer (512 channels)	52.6

## Conclusion

This paper addresses a significant challenge associated with the convolution operation, which often results in redundant calculations between channels when conducting inference and detection in image samples using convolution-based network models. With a specific focus on the identification and detection of Alligator Gar, the paper presents a novel approach. This approach introduces a lightweight network model for multi-scale detection, achieved through a fusion of involution and convolution, alongside the incorporation of a dual-channel attention mechanism.

Experimental results demonstrate that the method proposed in this paper excels across various detection metrics, whether applied to the specific task of Alligator Gar detection or general multi-type target detection tasks. Notably, the method significantly improves detection efficiency and reduces model size when compared to approaches like Faster R-CNN, SSD, and others. The detection speed of this method surpasses YOLOv5 (medium) by 48.4%, and the model size is only 76.5% of that of YOLOv5 (medium), in comparison to the latest State-of-the-Art (SOTA) method, YOLOv8(m), our method exhibits notable advantages in terms of lightweight design. This method offers substantial practical value, making it a promising contribution to the field of object detection.

## Data Availability

The source code for this method is available for download at the following URL: https://github.com/neemperor/ARD-Net.git.
